# Parry Romberg syndrome with localized scleroderma: A case report

**DOI:** 10.4317/jced.51409

**Published:** 2014-07-01

**Authors:** Mohsin Khan, Mubeen Khan, Raju Negi, Nikita Gupta

**Affiliations:** 1 M.D.S, Masters Of Dental surgery. Department of Orthodontics and Dentofacial Orthopaedics, Bangalore, Karnataka, India; 2 M.D.S, Professor and Head, Department of Oral Medicine and Radiology, Government Dental College and Research Institute, Bangalore, Karnataka, India; 3 Post Graduate Student. Department of Oral Medicine and Radiology, Government Dental College and Research Institute, Bangalore, Karnataka, India

## Abstract

Parry Romberg syndrome(PRS) is a rare acquired poorly understood neurocutaneous syndrome of unknown etiology characterized by slow progressive atrophic changes commonly affecting one half of the face. The exact incidence and etiology towards the syndrome remains unclear. Apart from the multifactorial etiology proposed, the possible primary cause is mainly attributed to the cerebral disturbance of the fat metabolism. The syndrome overlaps with “en coup de sabre” morphea, with an ill defined relationship existing between the two. Parry Romberg Syndrome is an invalidating lesion that may be associated with different neurological, cutaneous, ocular, dental and autoimmune abnormalities. This report presents one rare case of 22 years old female patient with Parry Romberg syndrome associated with localized scleroderma, accompanied by a brief review of literature with classical clinical, radiographic, histological findings and the treatment of progressive hemifacial atrophy.

** Key words:**Parry Romberg syndrome, progressive facial hemiatrophy, morphea, localized scleroderma.

## Introduction

Parry Romberg syndrome commonly known as progressive facial hemiatrophy is a very rare degenerative condition characterized by a slowly progressive but self limited unilateral atrophy of the face affecting variably the skin, subcutaneous fatty tissue, muscle, connective tissue and bone. First it was described by Caleb Hillier Parry [1825] and later on by Moritz Henirich Romberg [1846] who described it as a syndrome (1). Eulenberg coined the term “progressive facial hemiatrophy” in 1871 (2). Even after the condition being recognized for more than 150 years the exact incidence and etiology remains unknown. However immune mediated processes are primarily considered. Waternberg concluded that hyperactivity sympathetic nervous system, due to disturbed central regulation leads to trophism of underlying fat and subcutaneous tissue (3). Other possible factors include trauma, viral infections, endocrine disturbances, heredity and auto immunity are believed to be involved in the pathogenesis of the condition (4). The atrophy is insidious in onsfiget occurring in the first two decades of life lasting for 2-10 years followed by burning out of the atrophy with resultant stability (5). The condition is most often found in female population compared to male in the ratio 3:2 and has predilection for left side of the face. However 10% of the cases have been reported with entire half of the body involvement (6).

Unilateral facial involvement is common and generally follows the pattern of sensory innervations of one or all three trigeminal nerve dermatomes (7). The disease is usually limited below the forehead region though involvement of the scalp along with loss of hair or premature graying can also be seen. However 5-10% of cases being bilateral have also been reported (6). Parry Romberg syndrome is usually accompanied by neurological complications like trigeminal neuralgia, migraine, seizures and also accompanied by ocular complication of Horner’s syndrome (8). The relationship between linear scleroderma and progressive facial hemiatrophy is not clearly understood with some patients presenting a demarcation line between normal and abnormal skin, known as “coup de sabre” [French term which means “cut of the sword”] (9).

## Case Report

**Figure 1:**
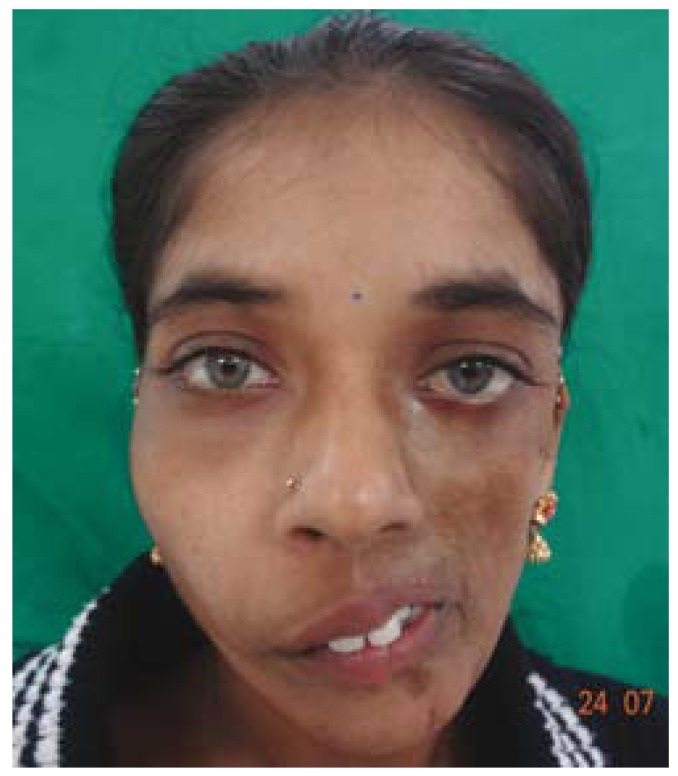
Photograph showing progressive atrophy of left half of the face.

**Figure 2:**
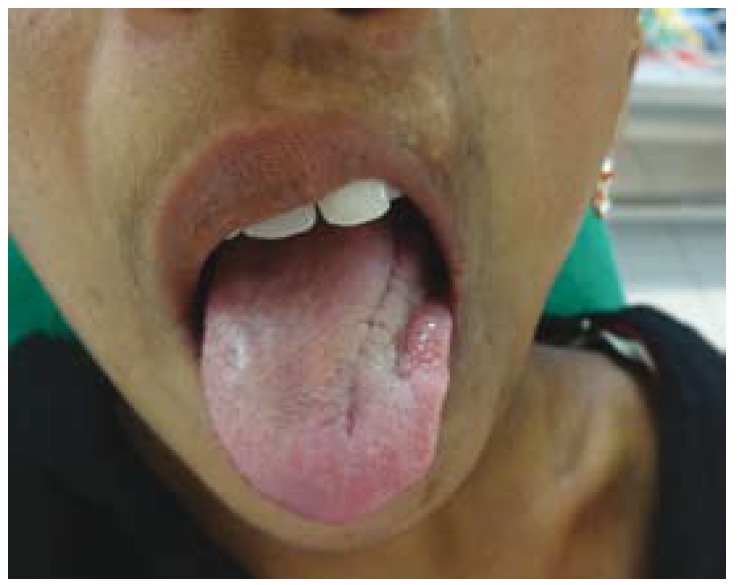
Intraoral Photograph showing marked atrophy of tongue on the left side with shift in the dorsal median fissure.

**Figure 3:**
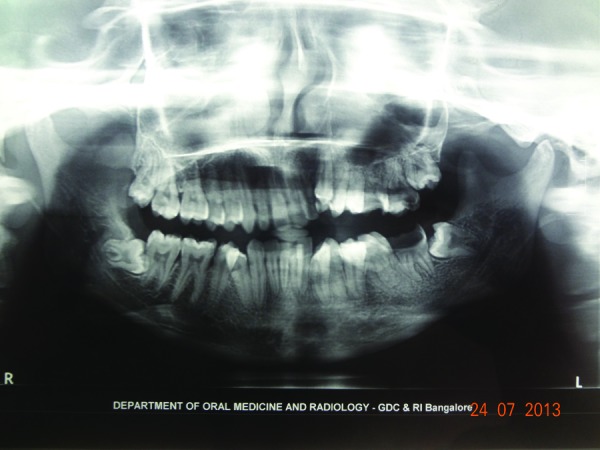
Panoramic Radiograph showing asymmetry with smaller condyle and coronoid process, short roots on affected side when compared to right side.

A 22 years old female patient reported to our department with a complaint of facial disfigurement on the left side of the face which gradually progressed over the past 14 years. Progressive atrophy on the left half of the face was initially noticed at the age of 8 years as an area of hyper pigmentation on the left malar area which gradually progressed with resultant atrophy of the underlying fat and subcutaneous tissue resulting in the present disfigurement (Fig. 1). However it was not associated with any other symptoms except for the limited mouth opening which the patient experienced gradually as the condition progressed. The patient had not sought any consultation earlier, for the facial disfigurement. Her medical and family history was non contributory. On general physical examination patient was conscious, oriented, a febrile, general condition was fair and vital signs were stable. Right side of the face appeared normal with marked facial asymmetry noted on left side. Head and neck examination confirmed the asymmetry with flattening, hyperpigmented, parchment like overlying skin. There was an evident loss of underlying fat, muscle and subcutaneous tissue giving a sunken in appearance. Thinning of the lips on the affected side with exposition of the teeth due to the corner of the mouth pulled to left side. Ocular examination and skin examination showed no abnormality with any evidence of lymphadenopathy. There was no evidence of sensory or motor deficits on both sides. On palpation, the skin on the affected side was rigid with signs of sclerosis. TMJ on the affected side was tender with deviation to the left on mouth opening. Intraoral examination revealed normal moist mucosa but tense oral mucosa on the affected side. Tongue on the affected side showed marked atrophy on the left side with shift in the dorsal median fissure (Fig. 2). Hard tissue examination revealed crowding of the teeth with shift in midline to the affected side and obliteration of the buccal and vestibular sulcus due to tense oral tissue. Upper and lower arch showed constriction of the arch with multiple carious teeth on left side. Based on history and clinical examination a preliminary diagnosis of hemifacial atrophy of the left side of the face was made. Routine blood investigation revealed values within normal range, Patient was negative for Anti nuclear Antibodies suggestive of absence of any auto immune disease. Biopsy specimen of the affected skin lesion showed sclerodermoid tissue reaction suggestive of morphic changes. Radiographic investigation of chest, lateral lumbosacral spine, terminal phalanges of long bone showed no abnormality. Orthopantamograph [OPG] revealed significant findings of asymmetry with thinning of the body of mandible with smaller condyle and coronoid process, short roots on affected side when compared to right side. Crowding and carious tooth was also noted (Fig.3). Paranasal sinus view showed hypoplasia of the frontal and maxillary sinus. Posterio- anterior view revealed asymmetry of the jaws. Computed tomography [CT] scan revealed generalized atrophy of the soft tissue of the left hemi face resulting in asymmetry on the left side with deviated nasal septum and marked hypoplasia of the frontal and maxillary sinus. Computed Tomographic scan of brain was normal.

Correlating all the investigation with the patient’s history and clinical examination a final diagnosis of Parry Romberg Syndrome was established. Dental and reconstructive plastic surgery using autologous fat transplant was considered as part of the treatment.

## Discussion

Parry Romberg syndrome is an uncommon degenerative and poorly understood condition. It is characterized by a slow and progressive unilateral atrophy of the facial tissues, including muscles, bones and skin. The condition is more often found in female population and has predilection for the left side of the face, which is seen in presented herein case. More than an aesthetic concern, this disease brings several functional and psychological problems due to asymmetry of the face. The extension of atrophy is frequently limited to on one side of the face, and the ipsilateral involvement of body is rare. In the case presented here; there was involvement of only one side of the face. The most important features of this disease is the dental involvement seen in 50% affected individual which includes deviation of mouth and nose to the affected side, and the unilateral exposition of teeth [when lips are involved] with dental involvement (10) . Clinically, the skin can be dry and hyper pigmented, which is seen herein presented case with dental involvement. Sometimes an ill defined conflicting relationship exists between PRS and linear scleroderma leading to the controversy and confusion over whether progressive facial hemiatrophy is a distinct disease or a form of linear scleroderma. Linear scleroderma is a subtype of localized scleroderma with unknown etiology that characteristically involves sclerosis confined to the skin. Differentiating the two, remains arbitrary as some patient initially begin with linear scleroderma and progresses to progressive hemifacial atrophy. However the syndrome is considered as a clinical subtype of linear scleroderma based on the recent literature and the classification of Mayo Clinic (9). Our case presented clinically and confirmed histopathologically the features of localised scleroderma affecting left half of the face. Ocular involvement seen in 10-35%, the most frequent manifestation is the enophthalmos, due to fat loss around the orbit, was not observed in our patient (5). The neurological complications seen in 45%, such as trigeminal neuralgia, facial paresthesia, severe headache and contra- lateral epilepsy can also be present, but were not diagnosed in our case (11). Differential diagnosis of localised scleroderma, hemifacial microsomia, Bell’s palsy, unilateral ankylosis, hemifacial hypertrophy and trauma or burn scar can be considered. Radiographically, the teeth of the affected individuals have short roots and appear small, when compared to the uninvolved side. The similar findings were present in our case. Goals of the therapy are restoration of the contour and symmetry of the face, management of complications associated with the atrophy. The treatment is usually based on reposition of adipose tissue that was lost due to atrophy. Autogenously fat grafts, cartilage grafts, silicon injections and prostheses, bovine collagen, inorganic implants and recently cell fat mixed with platelet gel are some alternatives to aesthetic correction of the atrophy (12). This case report documents the classical features of this rare entity, con-tributing towards the understanding of the poorly understood condition-Parry Romberg Syndrome.
